# Factors influencing trust in algorithmic decision-making: an indirect scenario-based experiment

**DOI:** 10.3389/frai.2024.1465605

**Published:** 2025-02-04

**Authors:** Fernando Marmolejo-Ramos, Rebecca Marrone, Malgorzata Korolkiewicz, Florence Gabriel, George Siemens, Srecko Joksimovic, Yuki Yamada, Yuki Mori, Talal Rahwan, Maria Sahakyan, Belona Sonna, Assylbek Meirmanov, Aidos Bolatov, Bidisha Som, Izuchukwu Ndukaihe, Nwadiogo C. Arinze, Josef Kundrát, Lenka Skanderová, Van-Giang Ngo, Giang Nguyen, Michelle Lacia, Chun-Chia Kung, Meiselina Irmayanti, Abdul Muktadir, Fransiska Timoria Samosir, Marco Tullio Liuzza, Roberto Giorgini, Omid Khatin-Zadeh, Hassan Banaruee, Asil Ali Özdoğru, Kris Ariyabuddhiphongs, Wachirawit Rakchai, Natalia Trujillo, Stella Maris Valencia, Armina Janyan, Kiril Kostov, Pedro R. Montoro, Jose Hinojosa, Kelsey Medeiros, Thomas E. Hunt, Julian Posada, Raquel Meister Ko Freitag, Julian Tejada

**Affiliations:** ^1^College of Education, Psychology, and Social Work, Flinders University, Adelaide, SA, Australia; ^2^Centre for Change and Complexity in Learning, University of South Australia, Adelaide, SA, Australia; ^3^Faculty of Arts and Science, Kyushu University, Fukuoka, Japan; ^4^Graduate School of Human-Environment Studies, Kyushu University, Fukuoka, Japan; ^5^Computer Science, Science Division, New York University Abu Dhabi, Abu Dhabi, United Arab Emirates; ^6^African Masters of Machine Intelligence (AMMI), African Institute for Mathematical Sciences (AIMS), Limbe, Cameroon; ^7^Higher Schools, Pedagogical Institute, Astana International University, Astana, Kazakhstan; ^8^Department of General and Biological Chemistry, Astana Medical University, Astana, Kazakhstan; ^9^Department of Humanities and Social Science, Indian Institute of Technology Guwahati, Guwahati, Assam, India; ^10^Department of Psychology, Alex Ekwueme Federal University Ndufu-Alike, Ebonyi, Nigeria; ^11^Department of Psychology, Faculty of Arts, University of Ostrava, Ostrava, Czechia; ^12^Department of Computer Science, Faculty of Electrical Engineering and Computer Science, Technical University of Ostrava, Ostrava, Czechia; ^13^Department of English, Hanoi University, Hanoi, Vietnam; ^14^College of Arts and Sciences, Notre Dame University, Cotabato, Philippines; ^15^Department of Psychology, National Cheng Kung University, Tainan, Taiwan; ^16^Department of Communication, University of Bengkulu, Bengkulu, Indonesia; ^17^Principles and Implication of Mind Sciences, Psychology Program, National Cheng Kung University, Tainan, Taiwan; ^18^Postgraduate Program of Basic Education, University of Bengkulu, Bengkulu, Indonesia; ^19^Library and Information Science, University of Bengkulu, Bengkulu, Indonesia; ^20^Department of Developmental Psychology and Socialization, Università di Padova, Padua, Italy; ^21^Department of Medical and Surgical Sciences, “Magna Graecia” University of Catanzaro, Catanzaro, Italy; ^22^School of Foreign Languages, University of Electronic Science and Technology of China, Chengdu, China; ^23^Department of Educational Psychology, University of Education of Weingarten, Weingarten, Germany; ^24^Department of Psychology, Marmara University, Istanbul, Türkiye; ^25^Faculty of Psychology, Chulalongkorn University, Bangkok, Thailand; ^26^Faculty of Public Health, University of Antioquia, Medellín, Colombia; ^27^Department of Cognitive Science and Psychology, New Bulgarian University, Sofia, Bulgaria; ^28^Research Centre for Cognitive Science, New Bulgarian University, Sofia, Bulgaria; ^29^Departamento de Psicología Básica 1, Universidad Nacional de Educación a Distancia (UNED), Madrid, Spain; ^30^Instituto Pluridisciplinar, Universidad Complutense de Madrid, Madrid, Spain; ^31^Centro de Investigación Nebrija en Cognición (CINC), Universidad Nebrija, Madrid, Spain; ^32^Department of Management, University of Nebraska Omaha, Omaha, NE, United States; ^33^School of Psychology, University of Derby, Derby, United Kingdom; ^34^Department of American Studies, Yale University, New Haven, CT, United States; ^35^Departamento de Psicologia, Universidade Federal de Sergipe, Saõ Cristóvão, Brazil

**Keywords:** algorithms, data, AI, trust, statistical literacy, explainability

## Abstract

Algorithms are involved in decisions ranging from trivial to significant, but people often express distrust toward them. Research suggests that educational efforts to explain how algorithms work may help mitigate this distrust. In a study of 1,921 participants from 20 countries, we examined differences in algorithmic trust for low-stakes and high-stakes decisions. Our results suggest that statistical literacy is negatively associated with trust in algorithms for high-stakes situations, while it is positively associated with trust in low-stakes scenarios with high algorithm familiarity. However, explainability did not appear to influence trust in algorithms. We conclude that having statistical literacy enables individuals to critically evaluate the decisions made by algorithms, data and AI, and consider them alongside other factors before making significant life decisions. This ensures that individuals are not solely relying on algorithms that may not fully capture the complexity and nuances of human behavior and decision-making. Therefore, policymakers should consider promoting statistical/AI literacy to address some of the complexities associated with trust in algorithms. This work paves the way for further research, including the triangulation of data with direct observations of user interactions with algorithms or physiological measures to assess trust more accurately.

## Introduction

“Incorrect. I am not an AI. My code name is Project 2501. I am a living, thinking entity that was created in the sea of information.”—Puppet Master (Ghost in the Shell)

The Fourth Industrial Revolution is characterized by the ubiquity of information and digital technologies. This revolution is epitomized by Artificial Intelligence (AI) and Machine Learning (ML), and at the heart of AI/ML are algorithms. Institutions, organizations and governments are using algorithms to cope with the vast amounts of information in these social sectors and to speed up and optimize decision-making processes (Engin and Treleaven, 2019). For example, the widespread use of algorithms in society was particularly demonstrated by the research undertaken to understand the global impact of COVID-19. During this crisis, algorithms played crucial roles across multiple domains: statistical algorithms were deployed to model virus fatality curves and study intervention effectiveness (Vasconcelos et al., 2020), while machine learning techniques supported molecular, medical, and epidemiological applications (Bullock et al., 2020). The successful deployment of algorithms in such high-stakes scenarios underscores both their growing importance in societal decision-making and the critical need to understand the factors that influence public trust in algorithmic systems. This evolution of algorithmic applications extends beyond public health emergencies to numerous other domains where decisions can significantly impact human lives and society. From surveillance systems monitoring public spaces to algorithms managing financial markets and predicting economic trends, these tools increasingly mediate high-stakes decisions across various sectors. The growing reliance on algorithmic decision-making in such consequential contexts necessitates a deeper understanding of their societal implications and reliability.

Algorithms help people to make decisions that have wider social implications; algorithms have transformative social power (Beer, 2017) when they are used to integrate complex data, such as the risk factors of homeless people (Toros and Flaming, 2018) or identifying the people with the greatest need in relation to different diseases (Burdick et al., 2020). The use of algorithms to aid decision making implies that there should be some confidence in their reliability. This raises a number of important questions. First, how much trust do people place in algorithms? More specifically, does trust depend on the context in which the algorithm is used? Is trust determined by knowing how the algorithm works? And is trust affected by an individual's cognitive abilities?

This study examines how trust in algorithms is affected by the societal relevance of the algorithm, the declared reliability of the algorithm, and the level of data literacy of the cogniser. First, the three key concepts of AI/ML, data and algorithms are defined. Second, it provides examples of the nature and use of algorithms in society. Third, the issue of explainable algorithms and trust is considered. Finally, the nature of the current study and the working hypotheses are outlined.

###  AI/ML, data, and algorithms

Broadly speaking, artificial intelligence (AI) is any type of technology that automates processes to solve problems that are usually associated with human intellectual capabilities (Ertel, 2017). More specifically, AI aims to solve problems and achieve goals with limited or no human supervision. A closely related term is machine learning (ML). Originally coined by Samuel (1959), ML can be defined as a collection of algorithms (mainly statistical and mathematical) to build computers capable of learning through experience (see Jordan and Mitchell, 2015). While the terms AI and ML are often used interchangeably, ML may be considered a more appropriate term than AI. Stereotypically, AI tends to be associated with rather unrealistic narratives depicting agents capable of human behavior (see Cave et al., 2018), and such examples are not yet feasible (also known as general AI). ML refers to algorithms designed to perform specific tasks in an automated way (also known as narrow AI) (Dawson et al., 2019).

**Figure 1:**
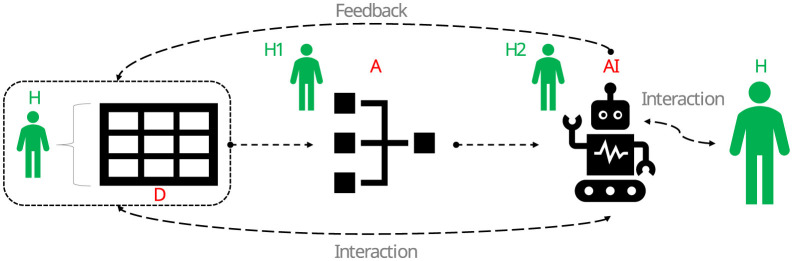
Relationship between data (D), algorithms (A) and artificial intelligence (AI) (ADA for short). Big data is used to feed algorithms, which in turn form the core of AI agents. There are four important aspects to note: (i) big data revolves (in one way or another) around human-related states, processes and events, (ii) such data is the substance of any algorithm, (iii) algorithms are the drivers of AI agents, and (iv) algorithmic/AI behaviors and outputs have implications for how new data is built and how humans (H) relate to ADA technologies in general. H1 and H2 are a subset of humans with specialized skills relevant to ADA. Source: the authors [icons from Font Awesome Free 5.2.0 by @fontawesome–https://fontawesome.com (https://commons.wikimedia.org/wiki/File:Font_Awesome_5_solid_robot.svg) and Mozilla (https://commons.wikimedia.org/wiki/File:Fxemoji_u1F6BB.svg)].

ML relies on data and algorithms (see Whittlestone et al., 2019), which together permeate many sectors of society (e.g., Schwab Intelligent Portfolios, Shanmuganathan, 2020). While algorithms can be defined as step-by-step procedures for solving a problem, data can be defined as numerical and categorical information about objects, events, processes and people that is digitally encoded (see Whittlestone et al., 2019). For example, the following steps represent a solution algorithm for estimating the central tendency in a vector of numbers: (i) sum all the numbers, and (ii) divide the result of the sum by the number of elements in the vector. This algorithm is known as the arithmetic mean (or average). The caveat of this algorithm is that it will be biased if the data does not follow a Gaussian shape. In other words, the output of this algorithm is only reliable if the data can be confidently shown to have a normal shape (e.g., via normality tests). In the context of AI-related technologies, algorithms are procedures designed to perform automated tasks using data sets to support human reasoning and decision making. In other words, data is used to feed algorithms, and algorithms in turn are used to drive AI agents (Siemens et al., 2022). Thus, algorithms are the “ghost in the shell” behind any AI agent. The Figure 1 illustrates this relationship between algorithms, data and AI (here ADA) (Whittlestone et al., 2019).

### The place of algorithms in society

Algorithms influence our daily lives. Whether it is defining our interests through our browser history (DeVito, 2017), determining what music we should listen to (Werner, 2020), or where we should go for dinner (Tenemaza et al., 2020). On a massive scale, algorithms are being used to extract information from so-called “big data” and support decision making in areas as diverse as surveillance (Tsakanikas and Dagiuklas, 2018), traffic management (Modi et al., 2021), and financial markets (Shanmuganathan, 2020). More recently, a new field of human-algorithm interaction mediated by natural language generation (NLG) systems has emerged, such as the Generative Pre-trained Transformer 3 model (better known as GPT-3) (Brown et al., 2020). GPT-3 produces human-like texts that are difficult to distinguish from texts written by humans (Clark et al., 2021), and this has begun to raise concerns about its use in various contexts, such as academic plagiarism (Dehouche, 2021) or computer programming (Ugli, 2020). While algorithms are increasingly embedded in our digital experiences, it is important to distinguish between their varying levels of impact on human lives.

As such, the majority of algorithms are used in a context that does not significantly affect our lives. We refer to these instances of algorithmic use as low-stakes scenarios. More recently, however, AI and ML algorithms have been used in scenarios that could have a significant impact. For example, algorithms are being used in hiring and promotion decisions (Drakopoulos et al., 2020), the criminal justice system (Rawat et al., 2021), and self-driving cars (Badue et al., 2021), to name a few. We call the latter a high-stakes scenario. That is, the above situations represent two types of scenarios in which algorithms could affect our daily lives: one with little involvement and almost no consequences (low-stakes scenario), and the other with great involvement and consequences (high-stakes scenario).

However, our interactions with algorithms are not limited to low-stakes and high-stakes scenarios and often involve preconceptions related to fear and distrust (Dwork and Minow, 2022). The literature suggests several explanations for why people do not trust algorithms, including a cost-benefit oriented logic where people tend to distrust algorithms even when presented with evidence of their superior performance, as they weigh potential risks more heavily than potential benefits (Debad, 2018). Many see algorithms as an “enigmatic technology” because they are difficult to understand (Beer, 2017) or in some cases, because people believe that algorithms are not capable of learning from their mistakes (Prahl and Van Swol, 2017), but at the same time they also believe that they could be replaced by computers (Granulo et al., 2019; Frank et al., 2019). Algorithmic bias can also affect trust [see examples in medicine (Vayena et al., 2018; Rajkomar et al., 2018). For a recent comprehensive report on trust in AI, see Gillespie et al., 2023].

“Technophobia”, a term coined by Rosen and Mcguire in the 1990s, describes the anxiety caused by a potential interaction with computers or computer-related technology, usually accompanied by negative attitudes toward computers (Rosen and Maguire, 1990; Kim, 2019). Recent demographic analyses have revealed nuanced patterns in technology anxiety. Research indicates no significant gender differences in technophobia scores between males and females, challenging earlier assumptions about gender-based technological comfort levels. The age distribution suggests that technophobia manifests across multiple generations, from young adults through middle age, rather than being concentrated among older populations as often assumed. Professional background data shows particular prevalence among educators and students, with experience levels primarily ranging from novice to moderate. While the studied sample was predominantly White, it also included smaller representations from other ethnic groups, such as Caucasian, Indian, and African American participants (Khasawneh, 2018; Kim, 2019). These findings suggest that technophobia's relationship with demographic factors is more complex than previously assumed, transcending traditional socio-demographic boundaries and affecting individuals across various social, professional, and cultural groups.

Similar existential fears dominate the public debate around concerns such as autonomous weapons (Human-AI Teaming, 2022; Warren and Hillas, 2020). One of these sociological fears is the fear of autonomous robots. This is a widespread fear in different countries (Liang and Lee, 2017; Gnambs and Appel, 2019), even though most people have not had contact with this type of robot. These fears could be the result of exposure to the way robots are portrayed in science fiction or social constructs related to robots, such as the possibility of being replaced by a robot at work (Liang and Lee, 2017; Gnambs and Appel, 2019). This polarization against robots and AI is fuelled by attention-grabbing events such as the recent confirmation by Blake Lemoine, a Google engineer, that the chatbox LaMDA has the ability to express thoughts and feelings like a human child (Luscombe, 2022) or the concerns about text generated by GPT-3 (Dale, 2021). These examples further distract the public from the most legitimate and worrying problems of these systems, such as “data colonialism” or the disturbing parallels between AI development and European colonialism (Adams, 2021). These parallels manifest in several ways: the extraction and exploitation of data from marginalized populations, mirroring colonial resource extraction; the use of Global South populations as testing grounds for AI systems developed in the Global North, reminiscent of colonial medical experimentation; and the imposition of Western conceptual frameworks of intelligence and ethics onto diverse cultural contexts. The field's emphasis on “ethics” often serves, paradoxically, as a form of technocratic rationalization similar to how ethical arguments were used to justify colonial expansion (Adams, 2021). Additionally concerning is that algorithms may reinforce preconceived stereotypes (Bender et al., 2021) and mishandle our personal data or who our data is shared with (Olhede and Wolfe, 2018), perpetuating historical patterns of discrimination and surveillance that characterized colonial governance. In addition, how the data given to algorithms is annotated has a direct impact on algorithmic performance (Tubaro et al., 2020), raising questions about whose worldview and categories are being encoded into these systems.

The media plays a significant role in shaping public perception of AI by covering two main sources of concern: autonomous technology and computer technology (Crépel et al., 2021). Autonomous technology refers to intelligent machines capable of making decisions independently, while computer technology encompasses software that supports communication and computation. The media tends to distinguish between these two categories and also differentiates between fear and criticism when discussing AI. This dichotomous approach to presenting the issues surrounding AI introduces a bias in how we perceive the risks associated with the technology. Consequently, this bias influences the level of trust we place in AI systems. The way the media frames the discussion about AI has a substantial impact on public opinion and can lead to a distorted understanding of the actual risks and benefits of the technology.

Developing a better understanding of how algorithms work and how to modify them can help reduce distrust in these systems, as suggested by several authors (Beer, 2017; Debad, 2018; Marmolejo-Ramos et al., 2022). When people have knowledge about how algorithms work, they can use this information to empower themselves as users. For example, music fans have acted collectively to boost the rankings of certain bands by engaging in massive streaming or downloading (Kang et al., 2022). Another example is Linkedln Brazil, which changed its algorithms to allow job ads targeted at Afro-Brazilians following social pressure (Milton Beck, 2022). These cases show that understanding how an algorithm works can both minimize suspicion and empower users. It is not necessary to understand all the technical details of how an algorithm works, but rather to understand that algorithms use statistical methods to classify, sort, rank and order information. This understanding of statistical concepts is called statistical literacy (François et al., 2020).

### Explainable algorithms

The knowledge required to understand and critically evaluate statistical results in order to make decisions based on them is defined as statistical literacy (SL) (François et al., 2020). Since its inception, the concept of SL has evolved (Haack, 1979) to include elements related to the context in which statistical reasoning can be applied (Wallman, 1993). SL plays a crucial role in society (Watson, 1997) and the communication of statistical information is now more important than ever (Gal, 2002). More recently, SL is leading individuals to recognize the importance of mathematics in the world (OECD, 2013).

Due to the statistical nature of algorithms, some level of SL is crucial to understanding what algorithms are capable of, but this understanding will also depend on the level of transparency or explainability of the algorithms (Friedrich et al., 2022). Explainability refers to the interpretability, comprehensibility or readability of the algorithm. Most of the latest algorithms are based on complex multi-layer networks, the basis of deep learning, which use an internal logic that experts cannot fully understand (Carvalho et al., 2019). These systems are called “black box” algorithms and various efforts have been made to promote their transparency (Du et al., 2019). Black box algorithms are less trusted than transparent models because they cannot be explained (Pasquale, 2016).

Several approaches have been proposed to increase the transparency of AI models and reduce systematic errors that affect their performance. One such approach is based on the concept of “model cards for model reporting” (see Figure 1 from Mitchell et al., 2019). This approach suggests that a comprehensive list of information should accompany the description of how the model was trained. This information should include details of the technician who developed the model, the intended use of the model, and the demographic or phenotypic groups on which the model has been tested. In addition, the model card should list the decisions made to optimize the model's performance and the various analyses carried out during the training process. Similar efforts to provide a framework for identifying biases associated with the data used to build or train AI models include the REVISE (REvealing VIsual biaSEs) (Wang et al., 2022) and The Spotlight (d'Eon et al., 2022) projects. These initiatives aim to increase transparency by systematically documenting and disclosing potential biases, enabling more informed use and interpretation of AI models.

Another more complex concern, also related to explainability, is the principle of explicability, a concept that combines intelligibility and accountability as the basis of an interpretable AI model (Herzog, 2022). The latter concept points to the importance of transparency, in the sense that all procedures and details used to build, train and test the AI model should be available during its development and use. This principle is part of the four principles endorsed by the OECD (2019) and the European Commission's High Level Expert Group on Artificial Intelligence (HLEG) to guide the development of “trustworthy” AI: respect for human autonomy, prevention of harm, fairness and accountability (Hagendorff, 2020). Despite consensus on these four principles, we are still far from creating a legal framework that guarantees accountability mechanisms in AI development (Mittelstadt, 2019).

In this context, our work presents an experimental study that looks at factors that might explain why people trust algorithms, such as: SL, explainability, stake levels, demographics, among others.

## Methods

### Participants

**Figure 2:**
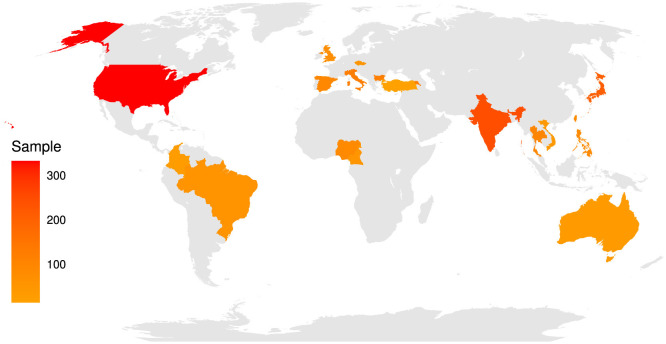
Geographical distribution of the sample participants. Armenia (females: 86, males: 40, age_*median*_ = 35.5, MAD = 14.08), Australia (females: 16, males: 16, age_*median*_ = 33.5, MAD = 14.08), Bulgaria (females: 101, males: 18, age_*median*_ = 21, MAD = 3.00), Brazil (females: 35, males: 24, age_*median*_ = 22, MAD = 4.40), Cameroon (females: 17, males: 35, age_*median*_ = 23, MAD = 5.93), Colombia (females: 18, males: 6, age_*median*_ = 25.5, MAD = 7.41), Czech Republic (females: 33, males: 18, age_*median*_ = 21, MAD = 1.48), Spain (females: 69, males: 23, age_*median*_ = 35.5, MAD = 14.08), Indonesia (females: 101, males: 28, age_*median*_ = 19, MAD = 0), India (females: 30, males: 82, age_*median*_ = 19, MAD = 0), Italy (females: 78, males: 43, age_*median*_ = 27, MAD = 5.93), Japan (females: 112, males: 86, age_*median*_ = 24, MAD = 4.45), Nigeria (females: 45, males: 40, age_*median*_ = 22, MAD = 2.97), Philippines (females: 66, males: 19, age_*median*_ = 20, MAD = 1.48), Thailand (females: 62, males: 30, age_*median*_ = 20, MAD = 1.48), Turkey (females: 9, males: 4, age_*median*_ = 23, MAD = 5.93), Taiwan (females: 59, males: 36, age_*median*_ = 20, MAD = 1.48), UK (females: 55, males: 17, age_*median*_ = 28, MAD = 11.12), USA (females: 142, males: 184, age_*median*_ = 22, MAD = 2.96), and Vietnam (females: 36, males: 2, age_*median*_ = 22, MAD = 0). 1% of participants had an elementary school education or less, 19% had a high school education, 13% had a post-secondary/non-tertiary education, 3% had an undergraduate education, 48% had a bachelor's education, 14% had a master's education, and 3% had a Ph.D. or higher education (see Supplementary files for details) (source: Wikimedia Commons, adapted from: https://commons.wikimedia.org/wiki/File:10-40_Window.svg).

Data from 3,260 participants were available from 20 countries (Armenia, Australia, Bulgaria, Brazil, Cameroon, Colombia, Czech Republic, Spain, Indonesia, India, Italy, Japan, Nigeria, Philippines, Thailand, Turkey, Taiwan, UK, USA, and Vietnam). However, only participants who provided complete data were included in the analyses (*n* = 1,921) (see Figure 2, M_*age*_ = 26.03 ± 9.88 SD; 59.5% women, 38.2% men, 1.8% other). Each participating laboratory obtained ethical approval from its local ethics committee, and data collection began only after ethical approval (the ethics approval for the leading research group in Australia was granted by the University of South Australia, with the approval number 203238. This approval was then used by the other participating laboratories to obtain their own respective ethics approvals). All participants voluntarily accessed the internet link for this study and agreed to participate after reading the information page and agreeing to take part. They were recruited via social media using convenience sampling.

### Materials

This online survey consisted of four sets of questions: (1) a demographic questionnaire in which participants were asked about their first language, country of residence, age, gender, level of education, level of familiarity with ADA (their level of familiarity with ADA was assessed using a visual analog rating scale (VAS) ranging from 0 [not very familiar] to 5 [very familiar] and using up to two decimal places); (2) a VAS rating scale version of the six-item ‘propensity to trust scale items' from Merritt et al. (2013), with a range of responses from 0 (strongly disagree) to 5 (strongly agree), using up to two decimal places; (3) a selection of 14 items (questions 2, 4, 9, 10, 12, 14, 18, 19, 27, 31, 34-37) from the 37-item Basic Literacy In Statistics (BLIS) scale (Ziegler and Garfield, 2018). The 14 items from the BLIS were chosen to cover different statistical concepts equally, i.e., items 2 and 4 relate to data production, items 9 and 10 to graphs, items 12 and 14 to descriptive statistics, items 18 and 19 to sampling distributions, items 27 and 31 to hypothesis testing, items 34 and 35 to the scope of conclusions, and items 36 and 37 to regression and correlation (these items are available in the supplementary material via the Qualtrics files). Finally, (4) 12 scenarios related to situations in which algorithms are used (half related to low-stake situations and the other half to high-stake situations). Each scenario was followed by two questions (see below), which were answered on a VAS rating scale from 0 (not at all likely) to 5 (very likely), using up to two decimal places. The results of expert judgement of these items are provided in the supplementary material. All phases of the study were programmed and distributed using Qualtrics™.

### Scenarios relating to algorithms used

Two scenarios were created to illustrate different situations in which people interact with algorithms. Half of them represented low-stake situations, i.e., (1) algorithms to make restaurant recommendations, (2) to select stories for online news, (3) to organize and sort emails, or (4) to suggest new restaurants, (5) new clothes, and (6) new music. The other half represented high-stakes situations, i.e., (7) algorithms to support court decisions based on psychological profiles, (8) to select CVs, (9) to make hiring recommendations for a job, (10) to select the best candidate for a position at a university, (11) to control the brakes of autonomous vehicles, and (12) to decide the priority of care in a medical context.

Each scenario contained a sentence related to its explainability. These sentences contained information about a specific machine learning method used by the algorithm (e.g., clustering learning methods, classification learning statistical methods, logistic regression methods, dimensionality reduction techniques, supervised statistical methods and clustering statistical methods). The sentence also briefly mentioned the quality of the method.

The following are examples of two different scenarios used to evaluate trusting algorithms:

#### Scenario 1—Low stake

**Overall context:** A new reservation app uses algorithms to make dining recommendations to its users, only revealing the three restaurants in the area available for a reservation that are the best match for your needs. The algorithm is based on information provided to the system by the user about restaurant preferences and requirements.
**With explainability:** The algorithm relies on clustering learning methods and has shown a high predictability accuracy across a variety of restaurants.
**Specific context:** You decide to use the app to find a recommendation for a dinner with your close friends next Friday. The app produces three restaurants with reservations available at the time you selected.
**Questions: 1**. How likely are you to regularly trust this app for decisions regarding restaurant reservations? 2. How likely are you to recommend this app for restaurant reservations to others?

#### Scenario 2—High stake

**Overall context:** A new employee selection software uses algorithms to make hiring recommendations to its users, only revealing the top candidates in the candidate pool that are the best match for the company's needs. The algorithm is based on information provided to the system about preferences and requirements for the job.
**With explainability:** The algorithm uses clustering statistical techniques and has shown high predictability when selecting candidates.
**Specific context:** You decide to use the software to find a recommendation for who to bring in for an onsite interview for an important role in your company. The software produces three recommended candidates who match the criteria.
**Questions: 1**. How likely are you to regularly trust this software for decisions regarding hiring? 2. How likely are you to recommend this software for hiring decisions to others?

### Procedure

**Figure 3:**
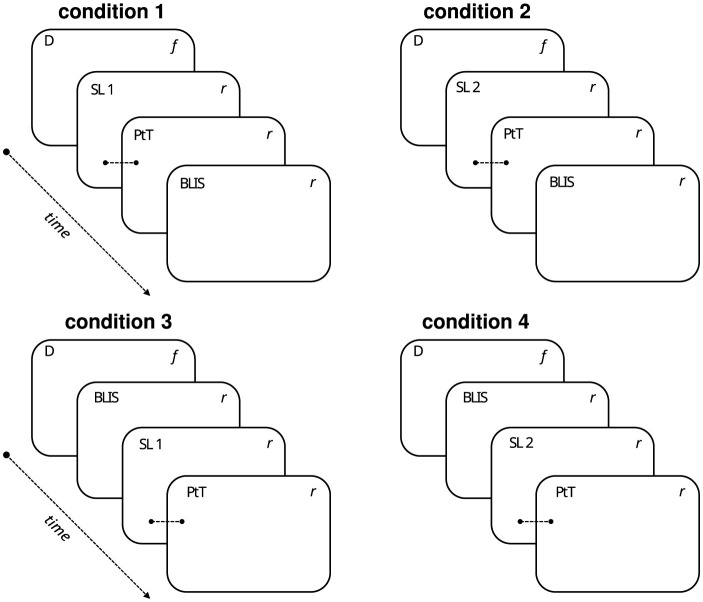
Illustration of the four experimental conditions to which participants were randomly assigned. D = demographic questions (age, gender, education level, open-ended question about what algorithms are, and VAS rating of participants' level of familiarity with ADA). SL = 12 scenarios list 1 and 2 (list 1 = six low-stake scenarios with explainability and six high-stake scenarios without explainability, scenario list 2 = six low-stake scenarios without explainability and six high-stake scenarios with explainability). PtT = six-item propensity to trust scale. BLIS = 14-item BLIS scale. *f* = Items were presented in a fixed order. *r* = items presented in random order. Note that PtT always followed one of the two scenario lists.

The experiment is a 2 × 2 factorial design: the importance of the situation in which an algorithm is used (low and high stake situation) and the explainability of the algorithm (with and without). These factors were implemented in the 12 scenarios via two lists; list 1 = six low-stake scenarios with explainability and six high-stake scenarios without explainability, and scenario list 2 = six low-stake scenarios without explainability and six high-stake scenarios with explainability. The four sets of questions were counterbalanced across participants, resulting in four experimental conditions (see Figure 3). Qualtrics ensured that participants were randomly assigned to each condition and that a balanced number of responses were collected for each condition. While the median time to complete the task was 24 minutes, there was some variation, with an interquartile range of 27 min (i.e., half of the participants completed the task within a 27-minute time span).

### Statistical analyses

Data analysis was conducted using multilevel linear models implemented in the R packages lmerTest and lme4 (Kuznetsova et al., 2015, 2017). The significance level for all statistical tests was set at α = 0.05. The model tested was: p~e*S*BLIS+*g*+a+ADA+c+(1|*id*) + (1|*i*) where “p” is the probability of trusting/recommending/using algorithms, “e” is the presence of explainability, “S” is the stake level (i.e., high and low stake), “BLIS” represents statistical literacy (frequency of correct answers), “g” represents participant gender, “a” represents participant age, “ADA” represents participant familiarity with ADA, “id” represents subject identification, “i” represents each of the 12 scenarios, and “c” represents participant country (“*” represents main effects and interactions. Only numeric variables are shown in teletype font; other variables are categorical. The variable “propensity to trust scale” was not added as a covariate as it showed a high correlation with the dependent variable, *r*_(1768)_ = 0.69, *p* < 0.001).

A stepwise backward model/variable selection algorithm was applied to this model to produce a significant and parsimonious model. The initial and final models were evaluated using metrics such as AIC and AICc weights (Wagenmakers and Farrell, 2004), *R*^2^ (coefficient of determination) for conditional (both fixed and random effects) and marginal (fixed effects) models, and performance score. These metrics were estimated using the performance R package (Lüdecke et al., 2021).

Once a parsimonious model was found, the marginal and conditional *R*^2^ values were estimated using the r2 nakagawa command from the performance R package (Lüdecke et al., 2021), then, the variance components of the random factors were estimated using the gstudy command from the gtheory R package (Huebner and Lucht, 2019).

For access to all materials and analysis codes, including a machine learning approach, visit the following link: https://figshare.com/projects/Trust_in_algorithms_An_experimental_approach_-_Data_repository/156212.

## Results

The stepwise backward evaluation suggested the same model as the initial model (see Section “Statistical analyses”). Tables 1, 2 provide a summary of the models, while Table 3 provides an ANOVA-like table for the model. An evaluation of the assumptions of the linear model using the R package gvlma showed that these assumptions were not met (Peña and Slate, 2006) (although, a QQ plot of the residuals showed no significant deviation from normality). As a result, a robust linear mixed model (Koller, 2016) was fitted using the robustlmm R package, and the estimates obtained were similar to those of the linear mixed model. These results are not unexpected, as previous research has shown that linear mixed models are robust to violations of distributional assumptions (Schielzeth et al., 2020). Further details of the statistical models can be found in the supplementary material.

**Table 1:** Fixed effects for the linear mixed model.

	**Estimate**	**Std. error**	**df**	**t value**	**Pr(>|*t*|)**	**Effect size (*d*)**
(Intercept)	1.466e+00	1.157e-01	3.354e+01	12.669	2.46e-14***	
eWITHOUT	6.231e-02	6.489e-02	2.567e+03	0.96	0.337013	
SLS	2.479e-01	3.527e-02	4.350e+04	7.03	2.10e-12***	0.217
BLIS	-6.020e-01	1.277e-01	2.457e+03	-4.714	2.57e-06***	-0.526
Age	-6.183e-03	1.444e-03	1.917e+03	-4.281	1.95e-05***	-0.005
Gender Male	-1.088e-01	2.541e-02	1.889e+03	-4.285	1.92e-05***	-0.095
ADA	5.483e-01	1.257e-02	1.895e+03	43.611	< 2e-16***	0.480
Country AU	-1.234e-01	1.018e-01	1.876e+03	-1.213	0.225469	
Country BG	-3.737e-02	6.830e-02	1.880e+03	-0.547	0.584321	
Country BR	8.501e-02	8.216e-02	1.878e+03	1.035	0.300939	
Country CM	-1.420e-01	8.690e-02	1.878e+03	-1.634	0.102445	
Country CO	1.063e-01	1.146e-01	1.877e+03	0.928	0.353747	
Country CZ	-1.467e-01	8.722e-02	1.878e+03	-1.682	0.092771	
Country ES	9.634e-03	6.978e-02	1.876e+03	0.138	0.890202	
Country ID	-8.746e-02	6.744e-02	1.880e+03	-1.297	0.194838	
Country IN	1.470e-02	7.303e-02	1.882e+03	0.201	0.840448	
Country IT	-2.432e-02	6.597e-02	1.989e+03	-0.369	0.712430	
Country JP	-2.351e-01	6.132e-02	1.880e+03	-3.833	0.000131***	-0.205
Country NG	-7.398e-02	7.471e-02	1.879e+03	-0.99	0.322211	
Country PH	-6.087e-02	7.539e-02	1.880e+03	-0.807	0.419529	
Country TH	-6.954e-02	7.514e-02	1.881e+03	-0.925	0.354832	
Country TR	1.614e-01	1.496e-01	1.877e+03	1.079	0.280707	
Country TW	-7.210e-02	7.538e-02	1.881e+03	-0.956	0.338971	
Country UK	-1.577e-01	7.606e-02	1.876e+03	-2.073	0.038301*	-0.138
Country US	-1.184e-01	5.752e-02	1.881e+03	-2.059	0.039599*	-0.103
Country VN	-1.397e-01	9.694e-02	1.878e+03	-1.441	0.149782	
eWITHOUT:SLS	4.463e-02	5.006e-02	4.353e+04	0.891	0.372671	
eWITHOUT:BLIS	2.067e-02	1.695e-01	2.565e+03	0.122	0.902951	
SLS:BLIS	1.225e+00	9.136e-02	4.349e+04	13.406	< 2e-16***	1.071
eWITHOUT:SLS:BLIS	-3.898e-01	1.302e-01	4.351e+04	-2.994	0.002755**	-0.341

The *R*^2^ values correspond to the Nagakawa coefficients (Nakagawa et al., 2017): $R_{cond}^{2}=0.363$ and $R_{marg}^{2}=0.241$. Country names are identified by the ISO 3166 standard. The reference category for the variable “gender” is female, and the reference category for the variable “country” is Armenia (AM). Effect sizes for significant variables were estimated following Brysbaert and Stevens (2018) (these values are interpretable as Cohen's *d*).

Signif. codes: “***” [0, 0.001] “**” [0.001, 0.01] “*” [0.01, 0.05], [0.05, 0.1].

**Table 2:** Random effects for the linear mixed model.

**Groups**	**Name**	**Variance**	**Std. dev**.
ID	(Intercept)	0.21380	0.4624
Item	(Intercept)	0.03076	0.1754
Residual		1.06489	1.0319

The variance explained by the random factors (estimated via the function gstudy in the gtheory R package) were: ID = 16.3% and Item 2.3%.

**Table 3:** Analysis of deviance table (type III Wald χ^2^ tests) for the fixed effects of the model with the best fit.

	**χ^2^**	**Df**	**Pr(> χ^2^)**
(Intercept)	160.5147	1	< 2.2e-16***
e	0.9221	1	0.3369223
S	49.4186	1	2.068e-12***
BLIS	22.2199	1	2.431e-06***
Age	18.3263	1	1.861e-05***
Gender	18.3575	1	1.831e-05***
ADA	1,901.9323	1	< 2.2e-16***
Country	47.7956	19	0.0002746***
e:S	0.7948	1	0.3726661
e:BLIS	0.0149	1	0.9029413
S:BLIS	179.7312	1	< 2.2e-16***
e:S:BLIS	8.9643	1	0.0027531**

Signif. codes: “***” [0, 0.001], “**” [0.001, 0.01], “*” [0.01, 0.05], [0.05, 0.1].

The intercept of the resulting mixed linear model was 1.46 (see Table 1), suggesting that on a scale of 0 to 5, the probability of trusting, recommending, or using algorithms in explainable and high-stake scenarios, as rated by young women with lower BLIS and ADA scores, was 29.32% ($\frac{1.46}{5}$). This probability significantly increased for low-stake scenarios (34.2%) or higher ADA scores (40.3%) and significantly decreased for higher BLIS scores (17.2%), older age (29.1%), or when the survey was answered by men (27.1%). Some countries showed a significant decrease in the likelihood to trust, recommend, or use algorithms, such as Japan (24.6%), the US (26.9%), and the UK (26.1%) (see Figure 6). Regarding the interactions between predictors, the likelihood of trusting, recommending, or using algorithms significantly increased for low-stake scenarios combined with higher BLIS scores (53.8%) and significantly decreased for scenarios without explainability combined with low-stake and higher BLIS scores (21.4%), always compared to the intercept (see Figure 5).

In terms of main effects, the results suggest a positive association between the likelihood of trusting/recommending/using algorithms and statistical literacy and familiarity with ADA, and a negative association between the likelihood of trusting/recommending/using algorithms and age. That is, the higher the level of statistical literacy, the higher the likelihood of trusting algorithms, and the higher the familiarity with ADA, the higher the likelihood of trusting algorithms. Also, the older a person is, the less likely they are to trust algorithms (although focused analyses indicated a slightly negative association between age and BLIS, such an association must be treated with caution as the number of observations decreases with increasing age). In terms of gender, it was found that participants who identified their gender as male were less likely to trust, recommend or use algorithms than those who identified their gender as female or other (this situation may be related to the fact that men have statistically significantly higher average levels of BLIS than women or “other”; see supplementary materials for details). Finally, only three countries showed a trend toward less reliance on algorithms, all of them highly industrialized countries (see Figure 6).

**Figure 4:**
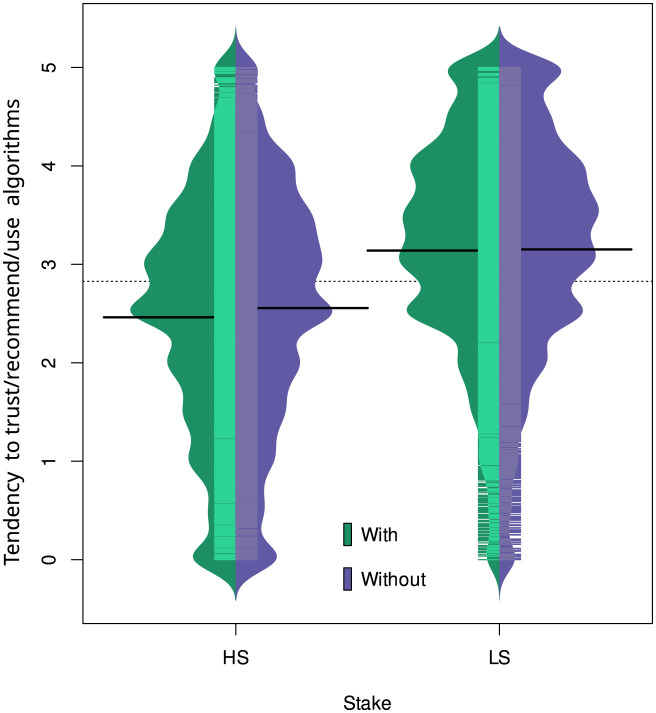
Beanplots showing the tendency to trust/recommend/use algorithms as a function of explainability (with or without) and situation stake (high stake = HS or low stake = LS). This figure shows the main effect of the stake factor (*S*) and the non-significant effect of explainability (*e*) (recall that this variable was not significant but used for illustrative purposes). The dotted horizontal line represents the grand mean and the four solid horizontal lines represent the groups' means.

**Figure 5:**
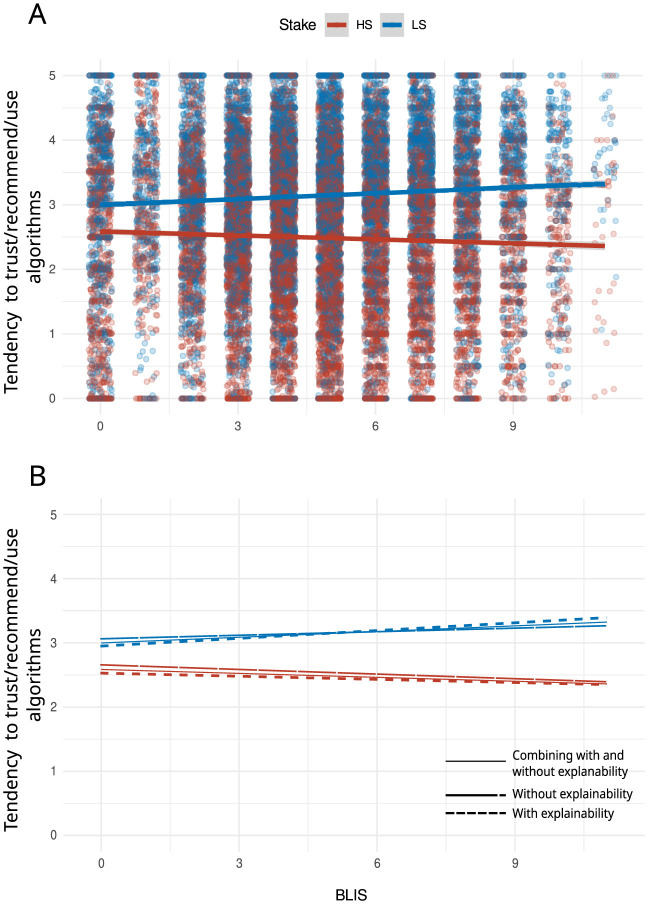
**(A, B)** Scatterplot showing the correlation between BLIS scores, explainability and the tendency to trust/recommend/use algorithms as a function of stake level. This figure illustrates the interaction between stake (high stake = HS or low stake = LS) and statistical literacy (BLIS) according to the level of explainability of algorithms (*e*). The observations on the *x*-axis are jittered for visualization purposes.

Figures 4, 5 show the main results in terms of the main effect of *S* and the two-way interactions between stake level (*S*) and statistical literacy (BLIS).

Figure 4 shows that the likelihood to trust/recommend/use algorithms is higher in low-stakes than in high-stakes scenarios, regardless of whether the scenarios have some explainability information or not. Figure 5 suggests that the likelihood to trust/recommend/use algorithms in low-stakes scenarios increases as the level of statistical literacy increases; however, in high-stakes scenarios, the likelihood to trust decreases as the level of statistical literacy increases.

## Discussion

The aim of this study was to investigate the personal characteristics (i.e., statistical literacy and demographics) and algorithmic characteristics (i.e., explainability and levels of stakes of algorithms) that influence people's trust in algorithms. The results showed a negative relationship between statistical literacy and trust in algorithms in high-stakes situations and a positive relationship in low-stakes scenarios. Explainability alone did not influence people's trust in algorithms. These results and their implications are discussed, as well as the limitations of the study.

Existing research has explored various factors influencing trust in AI. For instance, Lee (2018) highlighted the importance of perceived fairness of algorithms and users' perceptions of algorithm agency and intentionality. Araujo et al. (2020) investigated the roles of potential usefulness, fairness, and risk perceptions in users' engagement with algorithms. Cabiddu et al. (2022) examined factors such as users' inherent trust propensity and the drivers of information technology acceptance. Aysolmaz et al. (2023) focused on algorithm fairness, accountability, and privacy. Similar to the present study, some of these investigations employed fictional scenarios grounded in real-world contexts (Lee, 2018; Araujo et al., 2020; Aysolmaz et al., 2023), and one study utilized a comparable sample size of approximately 2,000 participants (Aysolmaz et al., 2023). Notably, none of these studies employed multicultural samples or examined the relationship between algorithm trust and statistical literacy. This gap was also identified in a systematic review by Mahmud et al. (2022), which encompassed over 80 empirical studies, none of which included statistical literacy as a factor influencing trust in AI.

This study is the first to examine the relationship between statistical literacy and trust in algorithms, revealing a nuanced relationship that depends on context. Our findings demonstrate that statistical literacy has opposite effects in different scenarios: it increases trust in algorithmic decisions for low-stakes situations while decreasing trust for high-stakes decisions. This differential effect suggests that statistical literacy enables a more sophisticated understanding of algorithmic capabilities and limitations. In low-stakes scenarios (such as restaurant recommendations or music suggestions), individuals with higher statistical literacy appear to recognize that algorithmic predictions based on pattern recognition and large datasets can be effective and reliable. However, in high-stakes contexts (such as employment or criminal justice decisions), this same statistical knowledge leads to greater skepticism - not because the algorithms are necessarily less accurate, but because statistically literate individuals better understand the potential consequences of algorithmic biases and limitations. Those with statistical literacy are better equipped to understand that while statistical models may achieve high average accuracy, they can still fail in critical individual cases or perpetuate systemic biases present in training data. This cautious approach to high-stakes algorithmic decisions reflects not just critical thinking, but a deeper understanding of how statistical methods work and where they may fall short.

Paradoxically, explainability only affected people's trust in algorithms when it was absent, the stakes were low, and statistical literacy was high. This contradicts previous findings in the literature, which have shown that interventions focused on explaining the decision-making processes of algorithms can increase the use of and trust in algorithms, for example in healthcare (Cadario et al., 2021), journalism (Shin, 2021) and military settings (Neyedli et al., 2011; Wang et al., 2011). One possible reason for this inconsistency could be due to the way we operationalized “explainability” in our study, where the explanations included technical jargon that may have exceeded the expected level of familiarity among participants. However, this may also mean that the information related to the explainability of the algorithm is not related to trust or distrust in the algorithm. Rather than focusing on how an algorithm works, our results suggest that statistically literate individuals primarily consider what the algorithm is being used for - its purpose and potential impact - when deciding whether to trust it. This finding challenges the common assumption that greater algorithmic transparency necessarily leads to more appropriate trust calibration.

Over time, the concept of statistical literacy has evolved from the understanding and application of statistical techniques to a broader understanding explicitly related to trust in algorithms. Algorithms now consist of thousands of lines of formulae and are increasingly used to make decisions that may be difficult for humans to understand (known as the black box effect). Consequently, statistical literacy now encompasses not only the ability to understand statistical output, but also the skills needed to critically interpret and evaluate statistical information and reasoning, which requires a higher degree of critical thinking. Therefore, the promotion of statistical literacy is essential to ensure that individuals have the necessary skills to understand and interpret statistical information and algorithms and to become critical users of ADA. Furthermore, our findings have important implications for policymakers and educators, who should consider incorporating statistical literacy training into school curricula and professional development programs. This can help ensure that individuals are equipped with the skills they need to navigate an increasingly data-driven world and make informed decisions based on statistical information and algorithms (but see Section “Implications and limitations” below).

Our results showed that older people and men were less likely to trust algorithms than younger people and women. Previous research has shown that certain demographic groups are more likely to trust algorithms than others. However, previous studies have shown that older people tend to trust ADA more than younger people, while gender has been shown to have inconsistent effects (see for example Hoff and Bashir, 2015; McBride et al., 2011). These differences may be due to particular characteristics of the study participants, possibly influenced by a bias toward certain aspects of the topic at hand.

**Figure 6:**
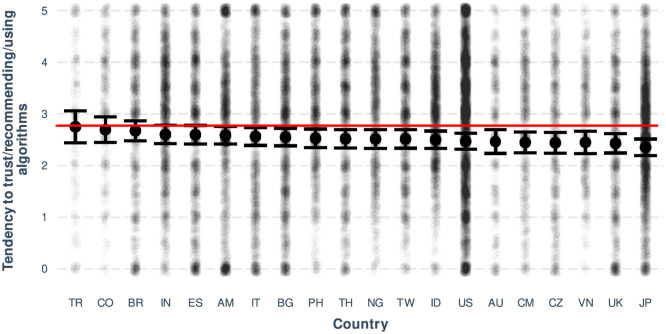
Plot showing the variability in the tendency to trust/recommend/use algorithms across countries. Countries are labeled with Turkey: TR, Colombia: CO, Brazil: BR, India: IN, Spain: ES, Armenia: AM, Italy: IT, Bulgaria: BG, Philippines: PH, Thailand: TH, Nigeria: NG, Taiwan: TW, Indonesia: ID, USA: US, Australia: AU, Cameroon: CM, Czech Republic: CZ, Vietnam: VN, UK: UK, and Japan: JP. The most important predictors for all models in each country were *S* followed by ADA and BLIS. Error bars represent 95% confidence intervals around the mean. The horizontal line indicates the overall mean. Although the substantial overlap of the confidence intervals suggests no significant statistical pairwise differences, the focus is on ranking countries based on their average tendency to trust algorithms.

In our cross-country analysis, we observed variations in trust in algorithms, with industrialized countries such as Japan, the US, and the UK exhibiting lower levels of trust in AI. This finding aligns with a recent study on trust in AI by Gillespie et al. (2023), which reported that Japan had one of the lowest levels of trust in AI, while the US and the UK had intermediate levels. Interestingly, countries such as India and Brazil, which demonstrated high levels of trust in the Gillespie et al. study (see Figure 2 in their report), appear in our linear mixed model with positive estimates (see Table 1, Figure 6), although not statistically significant. This suggests that different methodologies may yield varying perceptions of trust levels across countries.

## Implications and limitations

Various machine learning techniques require *data work* or human intervention in the form of data generation, annotation and algorithmic verification (Tubaro et al., 2020). This labor-intensive process is often distributed to teams in business process outsourcing companies (BPOs) or to individuals through labor platforms, reducing production costs (Casilli and Posada, 2019). Miceli and Posada (2022) studied one BPO in Argentina and three platforms operating in Venezuela and found that the discourses and social relations that structured data work were aimed at controlling workers (through managerial approaches in the BPO and algorithms in the platforms) to increase productivity and reduce worker “bias”. The problem is that feedback from workers was discouraged and, by taking clients' decisions as “ground truth”, the data production process reproduced clients' biases, which were carried out by algorithms trained on that data. Their research concluded that the quality of the data depended on the voice and engagement of workers, which in turn required decent working conditions and recognition. Even if the data used in the algorithm is well annotated and leads to good algorithmic performance, there is the question of the human ability to interpret these results, as human judgments are modulated by social-emotional processes (Schindler et al., 2015; Schindler and Kissler, 2016; Webb et al., 2022; Clark et al., 2021). Future work should consider the human and social aspects of data production and make the work visible in documentation efforts (Miceli et al., 2022). This transparency of the social aspects of datasets will contribute to trust in the operation of algorithms.

While the current findings are indeed informative, it is important to recognize certain limitations that may constrain the generalizability of these results and claims (Simons et al., 2017). We argued that statistical literacy influences trust in both low- and high-stakes scenarios; however, it could be part of a broader understanding of technology, algorithms, and data. Indeed, statistical literacy could be considered a sub-skill of AI literacy if AI literacy is understood as the ability to recognize, understand, use, and critically evaluate AI technologies and their societal impacts, supported by foundational knowledge in statistics and computing. Therefore, policymakers should consider promoting AI literacy to address some of the complexities associated with trust in algorithms.

Our study utilized self-reported measures via rating scales, which are efficient and cost-effective for capturing data on thoughts, feelings, and subjective experiences. However, these measures can be influenced by social desirability, response bias, misinterpretation, or lack of self-awareness. For instance, physiological research has shown that self-reported measures of physical activity can both overestimate and underestimate actual levels of physical activity (Prince et al., 2008). Therefore, future extensions of this work should consider a more robust approach, such as triangulating the data with direct observations of user interactions with algorithms or physiological measures to assess trust more accurately.

High-stakes and low-stakes situations exhibit significant variability across individuals and cultures, existing on a context-dependent continuum rather than as discrete categories. For example, choosing a restaurant for dinner with friends may carry different stakes across cultural contexts, socioeconomic backgrounds, and individual preferences. Our study's primary limitation lies in not systematically investigating how participants from different backgrounds interpreted and classified these scenarios. Additionally, while our sample included participants from 20 countries, certain geographical regions like Central Europe were underrepresented, potentially limiting the generalizability of our findings across different cultural contexts. Although we aimed to move beyond WEIRD (Western, Educated, Industrialized, Rich, and Democratic) sampling biases, more comprehensive geographic and cultural representation, along with larger sample sizes from each region, would be necessary to make broader generalizations about algorithmic trust across diverse populations (Nosek et al., 2022; Yarkoni, 2022). Future research should incorporate scenario validation across different cultural contexts and expand sampling to include currently underrepresented regions and demographic groups.

## Conclusion

This study investigated the personal and algorithmic factors that affect individuals' trust in algorithms. Our findings revealed that when the stakes are low, statistical literacy is positively correlated with the likelihood of trusting an algorithm. However, when the stakes are high, our results indicated a negative correlation between statistical literacy and the likelihood of trusting an algorithm. Therefore, we conclude that having statistical literacy enables individuals to critically evaluate the decisions made by ADA and consider them alongside other factors before making significant life decisions. This ensures that individuals are not solely relying on algorithms that may not fully capture the complexity and nuances of human behavior and decision-making.

## Author's note

An earlier version of this manuscript can be found at https://osf.io/preprints/psyarxiv/9wh2f. We recommend referring to and citing the current version rather than the earlier one.

## Data Availability

The datasets presented in this study can be found in online repositories. The names of the repository/repositories and accession number(s) can be found below: https://figshare.com/projects/Trust_in_algorithms_An_experimental_approach_-_Data_repository/156212.
